# Artificial Intelligence-Based Medical Data Mining

**DOI:** 10.3390/jpm12091359

**Published:** 2022-08-24

**Authors:** Amjad Zia, Muzzamil Aziz, Ioana Popa, Sabih Ahmed Khan, Amirreza Fazely Hamedani, Abdul R. Asif

**Affiliations:** 1 Department for Clinical Chemistry/Interdisciplinary UMG Laboratories, University Medical Center, 37075 Göttingen, Germany; 2 Future Networks, eScience Group, Gesellschaft für Wissenschaftliche Datenverarbeitung mbH Göttingen (GWDG), 37077 Göttingen, Germany

**Keywords:** text mining, artificial intelligence, machine learning, medical data, healthcare information

## Abstract

Understanding published unstructured textual data using traditional text mining approaches and tools is becoming a challenging issue due to the rapid increase in electronic open-source publications. The application of data mining techniques in the medical sciences is an emerging trend; however, traditional text-mining approaches are insufficient to cope with the current upsurge in the volume of published data. Therefore, artificial intelligence-based text mining tools are being developed and used to process large volumes of data and to explore the hidden features and correlations in the data. This review provides a clear-cut and insightful understanding of how artificial intelligence-based data-mining technology is being used to analyze medical data. We also describe a standard process of data mining based on CRISP-DM (Cross-Industry Standard Process for Data Mining) and the most common tools/libraries available for each step of medical data mining.

## 1. Introduction

With the rapid growth in online available medical literature, it is almost hard for readers to obtain the desired information without an extensive time investment. For example, in the ongoing COVID-19 pandemic, the number of publications talking about COVID-19 increased very rapidly. In the first 2 years of the pandemic, there were 228,640 articles in PubMed, 282,883 articles in PMC, and 7551 COVID-19 clinical trials listed in ClinicalTrials.gov databases (Data accessed on 16 February 2022), and this is increasing at an amazing speed. Because of the high degree of dimensional heterogeneity, irregularity, and timeliness, these data are often underutilized. This exponential growth in the scientific literature has made it difficult for the researchers to (i) obtain relevant information from the literature, (ii) present information in a concise and structured manner from an unstructured literature pile, and (iii) fully comprehend the current state and the direction of development in a research field.

The rapidly increasing literature cannot be managed and/or processed using traditional technologies and methods within an acceptable period. This massive volume of data makes it rather difficult for researchers to explore, analyze, visualize, and obtain a concise outcome. The process of extracting hidden, meaningful, and engrossing patterns from unstructured text literature is known as text mining [1]. Traditional text mining techniques are not sufficient to cope with the current large volumes of published literature. Therefore, a rapid increase in the development of new data mining techniques based on artificial intelligence can be seen on the horizon for the benefit of patients and physicians. The inclusion of artificial intelligence (also machine learning (ML), deep learning (DL), and natural language processing (NLP) as the subsets) empowers the data mining process with multifold benefits: Gaining new insights into the decision-making process, processing large dataset with increased accuracy and efficiency, and the ability to learn and improve continuously from the new data.

The current review sheds light on the role of different AI-based methods, i.e., NLP and neural network (NN) in medical text mining, the current data mining processes, different database sources, and various AI-based tools used in the text mining process along with various algorithms. We reviewed the latest text mining approaches, highlighted the key differences between medical and non-medical data mining, and presented a set of tools and techniques currently being used for each step of medical literature text mining. Additionally, we described the role of artificial intelligence and machine learning in medical data mining and pointed out challenges, difficulties, and opportunities along the road.

### 1.1. Medical vs. Non-Medical Literature Text Mining

Human medical data are unique and may be difficult when it comes to mining and analysis. First, due to the fact that humans are the most advanced and the most observed (in-depth) species on the globe, their observation is enriched because humans may provide their sensory input easily compared to the other species on the earth [2]. However, medical data mining faces numerous key challenges, mainly due to the heterogeneity and verbosity of data coming from various non-standardized patient records. Similarly, the insufficient quality of data is also a known issue in medical science that needs to be handled with care for data mining. Such challenges can be met by standardization of the process of selection of patients, collection, storage, annotation, and management of data [3]. However, sometimes this means that existing data and data acquired at multiple centers without good coordination and standard operating procedures (SOPs) could not be used. The major divergence between medical data and non-medical data mining is expected in ethical and legal aspects. The use of information that can be traced back to individuals involves privacy risks, which could result in legal issues. More than fifteen Federal US departments with the US Department of Health and Human Services have issued final revisions to the Federal Policy for the Protection of Human Subjects “the Common Rule, 45 CFR 46, Subpart A” (Protection of Human Subjects, 45 CFR 46 (2018). The federal framework for privacy and security does not apply to the information, which is de-identified or anonymized [4].

The ownership of medical data is another critical issue, as the data are acquired by different entities where the individuals may have been during their treatment or for diagnostic purposes. These entities can gather and store the data as per the authorization of the individual at the time of data acquisition. However, this permission on consent can be withdrawn by the patient at any time, and/or the consent is only valid for a limited period and data must be erased after this time [5]. Most of the clinical text is produced in a telegraphic way and the information is highly enriched. Additionally, it is written for the clinical staff and colleagues, therefore is full of incomplete sentences and abbreviations. Special tools are required to read, understand, and process this text [6]. Electronic patient records, also known as clinical text, have a unique problem in that they are written in a highly specialized language that can only be processed with a few available tools. Secondly, patient records are sometimes written in a telegraphic and information-dense style for clinician-to-clinician communication, and there exists no developed dictionary for such communications to check grammar and spelling mistakes. In addition, doctors and medical staff frequently use rudimentary sentences and frequently fail to mention the object, such as the patient, because the patient is implied in the text. “Arrived with 38.3 fever and a pulse of 132”, for example, could be written or simply mentioned.

### 1.2. Use of Artificial Intelligence and Machine Learning in Medical Literature Data Mining

The digital era has shown immense trust and growing confidence in machine learning techniques to increase the quality of life in almost every field of life. This is the case in health care and precision medicine, where a continuous feed of medical data from heterogeneous sources becomes a key enabler for AI/ML-assisted treatments and diagnosis. For instance, AI today can help doctors to bring better patient outcomes with early diagnosis and treatment plans as well as increased quality of life. Similarly, health organizations and authorities also aim for the timely execution of AI routines for the prognosis of outbreaks and pandemics at the national and international levels. Healthcare today is also witnessing the use of AI-aided procedures for operational management in the form of automated documentation, appointment scheduling, and virtual assistance for patients. In this section, we will see some real-life references of AI\ML tools and technologies currently used in various areas of medical sciences (Table 1).

Before going into further detail, it is worth mentioning that data mining and machine learning concepts go hand in hand and overlap each other to an extent but with a clear distinction of the overall outcome of both technologies. Data mining is the process of discovering correlations, anomalies, and new patterns in a large set of data from an experiment or event to forecast results [7]. The basis of data mining is statistical modeling techniques to represent data in some well-defined mathematical model and then use this model to create relationships and patterns among the data variables. Machine learning, on the other hand, is a one-step-ahead approach to data mining, where machine learning algorithms let the computer machine understand the data (with the help of statistical models) and make predictions of its own. That said, data mining techniques always require human interaction to find interesting patterns from a given dataset, whereas machine learning is a relatively modernized technique that enables computer programs to learn from the data automatically and provide predictions without any human interaction.

#### Natural Language Processing

Natural Language Processing (NLP) is an artificial intelligence (AI) discipline that converts human language into machine language. With the increased usage of computer technology over the last 20 years, this sector has grown significantly [8]. Clinical documentation, speech recognition, computer-assisted coding, data mining research, automated registry reporting, clinical decision support, clinical trial matching, prior authorization, AI chatbots and virtual scribes, risk adjustment models, computational phenotyping, review management and sentiment analysis, dictation and EMR implementations, and root cause analysis are some of the most popular applications of NLP in healthcare [9]. In the literature, a wide range of applications of NLP have been illustrated.

Liu et al. [10] used clinical text for entity recognition using word embedding (WE)-skipgram and long short-term memory (LSTM) techniques and achieved an accuracy of 94.37 percent, 92.29 percent, and 85.81 percent for de-identification, event detection, and concept extraction, respectively, based on the micro-average F1-score. Deng et al. [11] used concept embedding (CE)–continuous bag of words (CBOW), skip-gram, and random projection to generate code and semantic representations from clinical text. Afzal et al. [12] have developed a pipeline for question generation, evidence quality recognition, ranking, and summarization of evidence from biomedical literature and presented an accuracy of 90.97 percent. Besides these examples, Pandey et al. [13] listed 57 papers published between 2017 and 2019 that used NLP techniques and various text sources, such as clinical text, EHR inputs, Chinese medical text, cancer pathology reports, biomedical text, randomized controlled trial (RCT) articles, clinical notes, and EMR text-radiology reports, among others.

## 2. Standard Process for Data Mining

**Figure 1:**
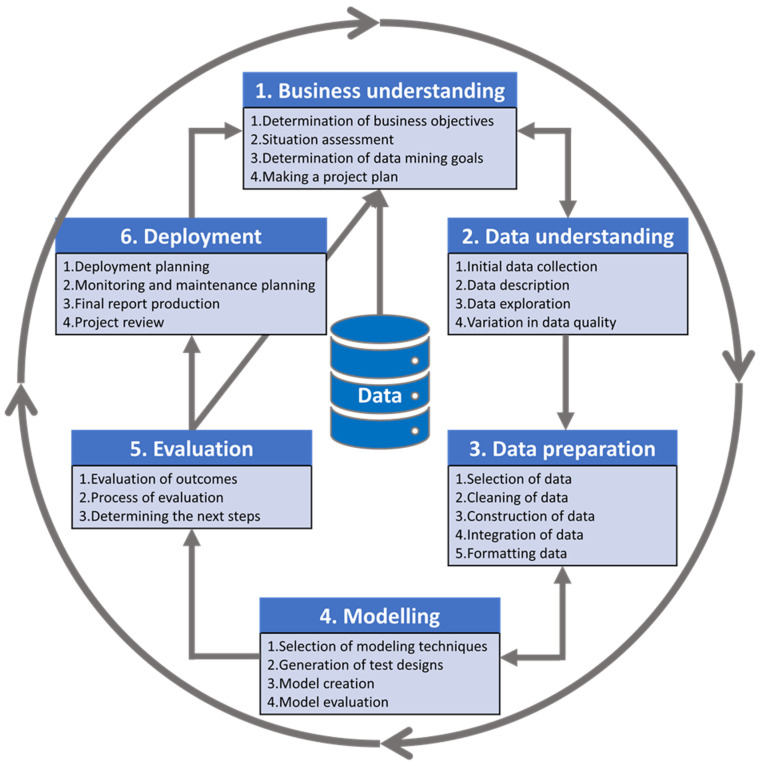
Cross-Industry Standard Process for Data Mining (CRISP-DM)—adapted from the webpage of the Data Science Process Alliance [17] (www.datascience-pm.com/crisp-dm-2/, accessed on 16 April 2022). The circular nature of the data mining process is symbolized by the outer circle, while the arrows that connect the phases show the most essential and common dependencies.

In response to the demand for a standard data mining method, industry leaders collaborated with a diverse group of practitioners (service providers, management consultants, data mining users, data warehouse vendors) and data mining experts to develop a free, well-documented, and non-proprietary data mining model [14]. Numerous methods are available for data mining, such as ASUM (Analytics Solutions Unified Method), CRISP-DM (Cross-Industry Standard Process for Data Mining), KDD (Knowledge discovery in databases), SEMMA (Sampling, Exploring, Modifying, Modelling, and Assessing), and POST-DS (Process Organization and Scheduling electing Tools for Data Science) [15]. In this study, we employ the CRISP-DM model for data mining because it is a complete and comprehensive data mining approach. In 1997, the CRISP-DM consortium developed a generic process model for data mining to establish guidelines for data mining beginners, the community, and experts, which can be modified for any particular need [14]. For example, to deal with the problem of multidimensional time-series data in a neonatal intensive care unit (NICU), the CRISP-DM model was modified to support and accommodate temporal data mining (TDM), which is named CRISP-TDM [16]. In the lifecycle of a data mining process, the CRISP-DM reference model has six phases (Figure 1): Business understanding, data understanding, data preparation, modeling, evaluation, and deployment. The details of the available tools and technologies for each phase are described in the rest of this article.

### 2.1. Business Understanding

The first and most critical part of data mining is business understanding, which includes setting project objectives, and targets, assessing the situation, execution plans, and risk assessments [14]. Setting project objectives requires a complete grasp of the project’s genuine goal to define the associated variables. The steps in the data understanding phase according to CRISP-DM are to (1) determine the business objectives (to fully comprehend the project’s goal, identify the key players, and establish business success criteria), (2) assess the situation (to identify resource availability (especially data), identify project risks and potential solutions to those risks, and calculate the cost–benefit ratio), (3) clarify the data mining goals (to establish project goals and success criteria), (4) produce a project plan (to develop detailed plans for each project segment, including a timeline and technology and tool selection).

Martins et al. [18] used a data mining approach to predict cardiovascular diseases (while using RapidMiner and Weka software). The main question addressed by the project is how to detect cardiovascular disease at an early stage in a person who is at a high risk of developing the disease and thus avoid premature death. As a result, the primary set of goals is to create a solution for predicting cardiovascular diseases in patients using patient data, to shorten the time required for disease diagnosis, and to provide the patients with immediate and adequate treatment.

### 2.2. Data Understanding

The emphasis in this phase (second phase), according to CRISP-DM, is on data source identification, data acquisition, initial data collection, familiarization with the data, and identifying problems in the acquired data. The steps in the data understanding phase are (1) acquire the initial data (to gather the data from various sources, insert it into the analysis program, and integrate it), (2) explain the data (to study and report on the acquired data’s surface properties such as field identities, data format, data quantity, and the number of records, etc.), (3) explore the data (to delve deeper into the data by querying, visualizing, and identifying relationships between data points, as well as to generate an exploration report), and (4) verify data quality (to inspect and document the data quality and any quality-related issues) [14]. In this phase, one focuses on identifying data sources for various types of data, the process of acquisition of the data, and handling access restrictions in data acquisition. A tremendous amount of data is generated by the health care industry and medical institutions every day from medical imaging, patient monitoring, and medical records [7]. Some of the most common types of medical data are experimental data, medical literature, clinical textual data, medical records, images/videos (e.g., MRI), and omics data (e.g., genomics, proteomics). For example, Martins et al. [18] used a data mining approach to predict cardiovascular diseases. For data understanding, the dataset for cardiovascular disease prediction came from the Kaggle data repository and focused on detecting cases of cardiovascular disease. The dataset included 70,000 registered patients with 12 disease-related attributes collected during the patients’ medical examinations.

#### 2.2.1. Literature Extraction/Data Gathering

The first task in the data understanding phase is to identify data sources, acquire data from these sources, identify problems during data acquisition, such as data restrictions and data privacy policies, and document the solutions [14]. Text/data mining frequently uses public Internet-based sources such as the World Wide Web. The retrieval of content from public sources is referred to as “web scraping” or “web crawling”. Web scraping can be performed manually, but it can also be performed automatically with the help of a web crawler. Manual scraping a large database such as PubMed, which contains millions of peer-reviewed publications, requires a lot of time and effort. Only automated processing can provide the necessary quality, response time, and homogeneity for their analysis with such a large database. As a result, there is always a high demand for web scraping techniques and tools tailored to customer requirements. PubMed, for example, is a massive database of biomedical literature that contains 34 million citations (as of 11 May 2022) collected from online books, life science journals, and MEDLINE, and a massive number of new publications are added every year [19]. Web crawlers are used to search for and harvest the necessary data from it. Guo et al. [20], for example, collected COVID-19 data published by local health authorities using a web crawler (developed using the Python language and connected with a MySQL database).

**Figure 2:**
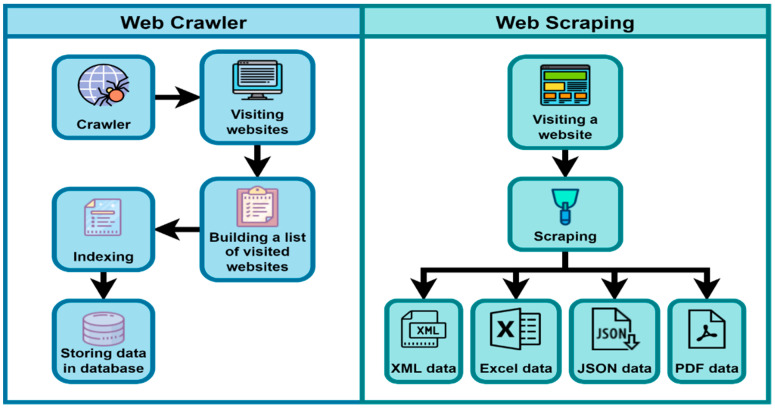
Comparison between web crawling and web scraping.

Although web scraping and web crawling may seem to be identical, they have several distinctions (Figure 2). While the terms “web scraping” and “web crawling” are sometimes interchanged, they refer to two distinct processes [21,22]. Web crawling is a broad term that refers to the process of downloading information from a website, extracting the hyperlinks included within, and following them (Figure 2). Typically, downloaded information is saved in a database or indexed to enable searching. Essentially, search engines are crawlers. All that is required is to see a page in its entirety and indexing it. When a bot crawls a website, it scans each page and link, all the way to the website’s last line, looking for any information. Web crawlers are primarily used by major search engines such as Google, Bing, and Yahoo, as well as statistics organizations and online aggregators. Typically, a web crawler collects general information, while scrapers collect particular datasets [23,24]. On the other hand, web scraping is the process of obtaining data from a web page and extracting specific information that can be saved almost anywhere (database, file, etc.) as shown in Figure 2. An online scraper, also known as a web data extractor, is similar to a web crawler in that it detects and locates website content. In contrast to a web crawler, which uses pseudo-random IDs, web scraping uses specific identifiers, such as the HTML structure of the web pages from which data must be collected. Web scraping refers to the use of robots to extract specific datasets from the internet. The obtained data can be compared, checked, and analyzed in accordance with the demands and objectives of a specific organization [25].

Several text mining tools are now available. Kaur and Chopra [26] compared 55 popular text mining tools and their features and discovered three categories: (1) Proprietary (company-owned—39 tools); (2) open source (free—13 tools); and (3) online text mining tools (run directly from a website—3 tools). Four tools that were not examined in the prior review but are now on the list of well-liked text mining tools are contrasted in Table 2. All of these Python-based tools serve the same purpose, but with different goals and objectives. ‘’Requests’’ has an advantage over other tools in that it is easy to use, making it an excellent choice for any simple web scraping task. Scrapy is best suited for large-scale web scraping projects, as opposed to the other three tools (requests, beautiful soup, and selenium), which are best suited for small-scale scraping tasks. The “Beautiful Soup” tool has advantages such as being simple to understand, learn, and use, and it can extract information from a disorganized website. Selenium has a significant advantage over the other scraping tools described because it can scrape websites with heavy JavaScript. Table 2 provides descriptions of more hierarchical comparisons.

##### Access Restriction

**Figure 3:**
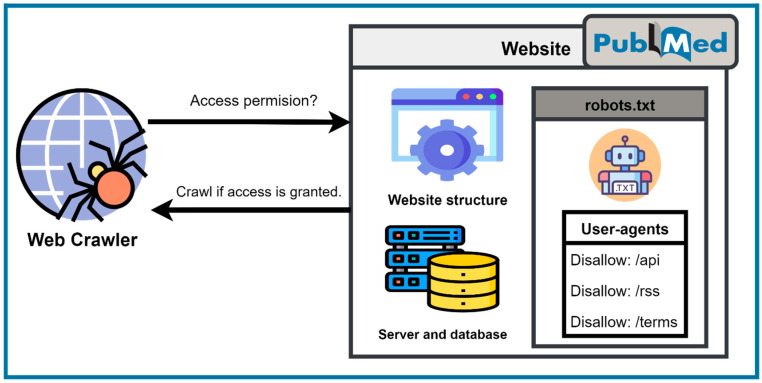
Layout for access restrictions.

When a web crawler visits a website, some pages or the entire website possess access restrictions. These restrictions are implemented mainly by the site owners due to data confidentiality, data integrity, and data quality, as well as legal concerns. A crawler usually performs multiple requests per second and downloads large files to obtain the data in a short time, which can cause a website server to crash. To tackle this problem, numerous methods are available. Canonical tag, robots.txt, x-robots-tag, the metarobots tag, and others are files provided by the website owners to follow the instructions for scraping the website without creating any problem. For example, “robots.txt” files are frequently used by websites to convey their scraping and crawling intents. Robots.txt files enable scraping bots to crawl specific sites, while malevolent bots, on the other hand, are uninterested in robots.txt files (which act as a “do not enter” sign) as explained below in Figure 3.

##### Data Collection from Different Sources

The pace at which medical data are being generated is increasing day by day during the massive information explosion year, and global information is being produced in massive quantities in every field, including healthcare [31,32]. Administrative records, biometric data, clinical registration, diagnostics, X-rays, electronic health records, patient report data, treatments, results, and other types of medical data are all included in medical data. These massive and complex characteristics make data difficult to deal with for a meaningful and unknown outcome. Healthcare centers and medical institutions around the world have proposed a variety of medical information systems to deal with rapidly growing data and provide the best possible services and care to patients [32]. The most common way to collect and store the data is by management software, which can store all electronic and non-electronic records. Several software products are available, e.g., eHospital Systems (adroitinfosystems.com/products/ehospital-systems, accessed on 11 April 2022) and the DocPulse Clinic/Hospital Information Management System (docpulse.com, accessed on 11 April 2022).

For text mining, data collection from data sources is the key step. In medical science, various types of medical data, as well as trends, are generated at a rapid pace, which can be differentiated into five categories, as follows:

1. Hospital management software (Patient data/Clinical narratives).
2. Clinical trials.
3. Research data in Medicine.
4. Publication platforms for Medicine (PubMed, for instance).
5. Pharmaceuticals and regulatory data.

Table 3, Table 4 and Table 5 provide further details about the different types of data sources. Patient data generated by clinical trials is available from various sources, as shown in Table 3. Medical researchers benefit from open-access databases because they have enormous volumes of data, rich data content, broad data coverage, and a cost-effective study strategy. There exist several datasets and databases publicly available related to various medical fields that contain many medical record variables (Table 4). Textual information is growing rapidly, and it is difficult to grab concise information fast and structured manner. The published literature is the most abundant and primary source of textual information in the health care field (Table 5).

### 2.3. Data Preparation

In the third phase (data preparation) of CRISP-DM, a final dataset is created from the raw data, which will be used in the modeling tool. This phase is the major part (ca. 80%) of a text/data mining project. The steps in the data preparation phase are (1) data selection (to choose the dataset along with its attributes that will be used for the analysis based on the project goals, quality, data type, and volume.), (2) data cleaning (to estimate missing data and improve the dataset by correcting, imputing, or removing incorrect values), (3) data construction (to create derived attributes or entirely new records, as well as to transform data as needed), (4) data integration (to create new datasets and aggregate new values by combining data from multiple sources), (5) data formation (to remove inappropriate characters from the data and change the data’s format or design so that it fits into the model) [14].

#### 2.3.1. Data Cleaning/Data Transformation

**Figure 4:**
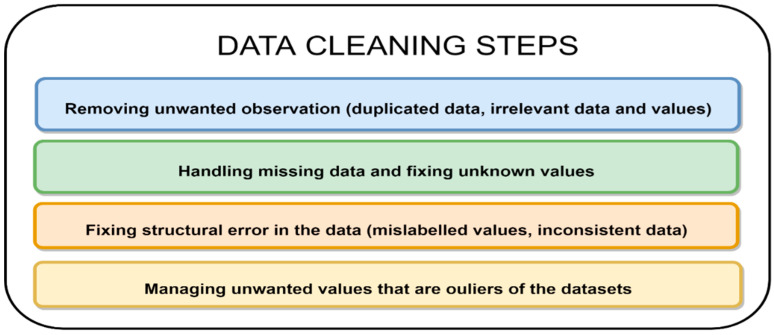
Steps for data cleaning.

The primary goal of data cleaning is to detect and remove duplicate data and errors from a dataset to create a reliable dataset. Cleaning data entails identifying and removing entries from a dataset that are corrupt, incorrect, duplicated, incomplete, or improperly formatted (see Figure 4). Data cleaning is required to analyze information from multiple sources [45,46,47].

Various related tools and python libraries are discussed in the following sections.

Python Libraries for Data Cleaning include the following:

1. NumPy is a quick and easy-to-use open-source Python library for data processing. Because many of the most well-known Python libraries, including Pandas and Matplotlib, are based on NumPy, it is a fundamentally crucial library for the data science environment. The primary purpose of the NumPy library is the straightforward manipulation of large multidimensional arrays, vectors, and matrices. For numerical calculations, NumPy also offers effectively implemented functions [48].
2. Data processing tasks such as data cleaning, data manipulation, and data analysis are performed using the well-known Python library Pandas. The Python Data Analysis Library is referred to as “Pandas”. Multiple modules for reading, processing, and writing CSV, JSON, and Excel files are available in the library. Although there are many data cleaning tools available, managing and exploring data with the Pandas library is incredibly quick and effective [49].
3. An open-source Python library for automating data cleaning procedures is called DataCleaner. Pandas Dataframe and scikit-learn data preprocessing features comprise its two separate modules [50].

The data are then transformed into the proper format after being cleaned (Excel, JSON, or XML). Data transformation makes it simpler to preprocess data and/or text. Depending on the modifications that must be made, the data transformation may be straightforward or complicated. The data are easier to use for both humans and computers after transformation because it is more structured and organized. Additionally, it becomes simpler to integrate into various programs and systems [46].

Various related tools are discussed in the following sections.

1. Generation of Bibliographic Data is known as GROBID. It is a machine-learning library that has developed into a state-of-the-art open-source library for removing metadata from PDF-formatted technical and scientific documents. The library plans to reconstruct the logical structure of its original document in addition to simple bibliographic extraction in order to support large-scale advanced digital library processes and text analysis.

GROBID develops fully automated solutions based on machine learning models for that reason. ResearchGate, Mendeley, CERN Inspire, and HAL, France’s national publication repository, are just a few of the commercial and open-access scientific services that the library is connected to.

The result is to extract and transform PDF documents into XML TEI format, supplement the extracted information with other online services, and illustrate the findings gathered in PDF documents of scientific papers [51,52].

2. BioC is a straightforward and straightforward format for exchanging text data and annotations, as well as for simple text processing. Its primary goal is to provide an abundance of research data and articles for text mining and information retrieval. They are available in a variety of file formats, including BioC XML, BioC JSON, Unicode, and ASCII. These formats are available through a Web API or FTP [53].

To summarize, data cleansing improves a dataset’s consistency, while transformation simplifies data processing. Both processes improve the training dataset’s quality for model construction.

#### 2.3.2. Feature Engineering

Choosing, modifying, and converting raw data into features that may be utilized in supervised learning is a process of feature engineering, often referred to as feature extraction. This machine learning technique, feature engineering, uses data to generate new variables that are not present in the training set. To streamline and accelerate data transformations while also improving model accuracy, it can generate new features for both supervised and unsupervised learning. With machine learning models, feature engineering is necessary. Regardless of the architecture or the data, a bad feature will directly affect your model. Numerous tools are available to automate the entire feature engineering process and to generate a large pool of features in a short period for both classification and regression tasks. Some feature engineering tools are FeatureTools, AutoFeat, TsFresh, Turi, Azure Machine Learning Studio, ZOMBIE, FeatureFu, and OneBM [54,55].

Vijithananda et al. [56] extracted features from MRI ADC images of a brain tumor. The following features were extracted from labeled MRI brain ADC image slices from 195 patients: Skewness, cluster shade, pixel values (he demographics), prominence, Grey Level Co-occurrence Matrix (GLCM) features, energy, contrast, entropy, variance, mean, correlation, homogeneity, and kurtosis. Both GLCM homogeneity and skewness were excluded because they scored the lowest in the ANOVA f-test feature selection process. The Random Forest classifier outperformed Decision Trees, Nave Bayes, Linear Discriminant Analysis, K-Nearest Neighbors (KNN), and Logistic Regression and was chosen for further model development. The final model had an accuracy of 90.41 percent in predicting malignant and benign neoplasms.

#### 2.3.3. Searching for Keywords

The extraction of keywords or key phrases from text documents is known as keyword extraction. They are chosen from among the phrases in the text document and describe the topic of the document. Several popular methods are available for automatically extracting keywords. Those are used in processes that automatically extract keywords from documents to select the most frequently used and significant words or phrases from the text document. This classifies keyword extraction methods as part of the natural language processing field, which is important in machine learning and artificial intelligence. [57]. Keyword extractors are used to extract words (keywords) or groups of two or more words that form a phrase (key phrases).

FlashText, for example, is a free and open-source Python package that enables keyword search and replacement and is one of the recently described keyword extraction tools [58]. It performs a full analysis using an Aho-Corasick algorithm and a Trie Dictionary. As a general rule, keyword matching entails scanning the corpus (human-created documents comprise a large, structured set of texts) for each term. Consider the following scenario: Someone has 100 keywords and needs to search through 2000 papers; a single term is selected at a time and a search of the 2k corpus is performed; the search is continued for 100 × 2000 is 200,000 iterations. In addition to this keyword search tool, four Python-based tools are selected from the various keyword and phrase extraction tools that are available, and their features, benefits, and NLP tasks are contrasted in Table 6.

### 2.4. Modeling

In the fourth phase (known as modeling) of CRISP-DM, various modeling techniques are tested and calibrated by adjusting the model parameters to achieve the best results [14]. The steps in the modeling process are (1) choosing a modeling technique to select one or more task-specific models/algorithms/assumptions, (2) the creation of test designs to determine the model’s strength by evaluating the model’s quality and validity, (3) the building of models (to use the modeling tool for building models from the prepared dataset, adjust the model parameter, and describe the model), and (4) the evaluation of models to explain the model outcome based on subject knowledge, the predetermined success norms, and the test design, rank the multiple generated models, and readjust the parameter settings—if required.

**Figure 5:**
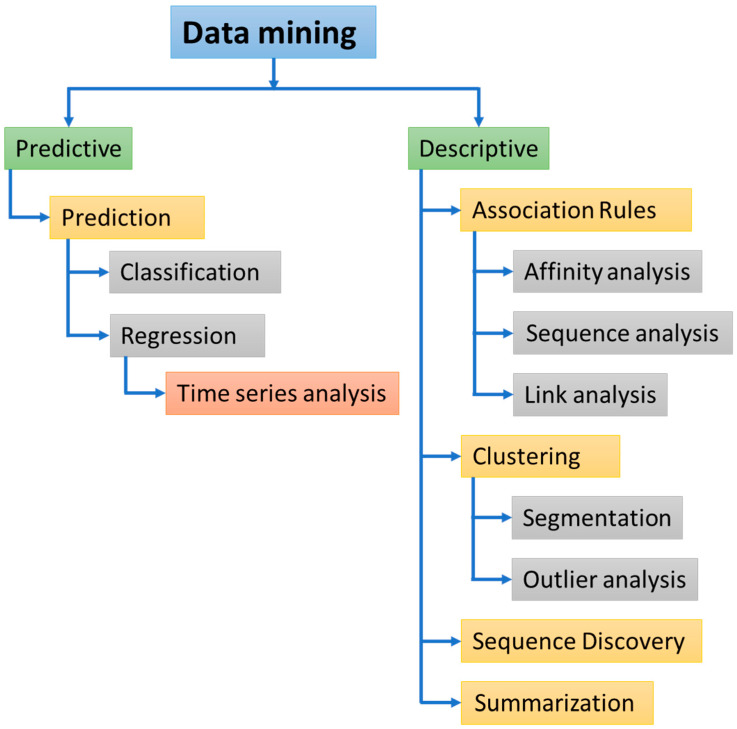
Predictive and descriptive data mining tasks.

From several available models for organizing and analyzing the data, the selection of a model depends on the purpose (e.g., forecast) and the type of data used (unstructured or structured). A model is a set of data, patterns, and statistics. The available data-mining models are divided into two categories: Predictive and descriptive. Descriptive models are frequently used to determine patterns in data that can be explained by humans. Predictive models use known results from various datasets to forecast unidentified or future values of other variables of interest. Predictive models are usually based on the previously provided data and their results. Classification, prediction, regression, and time series analysis are tasks in the predictive models. Descriptive model data mining tasks comprise clustering, associating rules, sequence discovery, and summarization (Figure 5). A number of algorithms/methods are available for the prediction and analysis of patterns in the data. However, the selection of the algorithm is mainly depending on the dependent variables whether labeled or unlabeled. If the dependent variable/s in the dataset are labeled, a supervised learning algorithm is used. Decision trees, the random forest (RF), support vector machines (SVMs), and competitive risk model are commonly used algorithms. In contrast, if the dependent variables in the data are not labeled, an unsupervised learning method is used. Clustering analysis, partition clustering, hierarchical clustering, principal component analysis (PCA), and association analysis are some of the unsupervised learning algorithms [64,65].

The dataset is the primary distinction between supervised and unsupervised machine learning. It is referred to as supervised learning if the dataset employs a labeled dataset for input and output, whereas unsupervised learning techniques use unlabeled data. As the name suggests, supervised learning entails the external supervision of a model’s training. Unsupervised learning, on the other hand, does not involve any supervision. Additionally, in the case of supervised learning, the goal is to predict the outcome of new data. In the case of unsupervised learning, the goal is to find hidden patterns and gain insight from enormous amounts of new data. In contrast to supervised learning models, which are straightforward, unsupervised learning models require a large training set to produce the desired results, making them computationally complex. Some of the applications of supervised learning models include diagnosis, identity fraud detection, image classification, price predictions, sentiment analysis, spam detection, market forecasting, and weather forecasting. Unsupervised learning models are used in the pipelines for anomaly detection, big data visualization, customer personas, feature elicitation, recommended systems, structure discovery, and targeted marketing [64,66].

As an instance of the modeling example, the suitability of a WebCrawler (StormCrawler) for the acquisition of all health-related web content on the German Health Web (Germany, Austria, and Switzerland) was investigated by Zowalla et al. [67]. For this purpose, a support vector machine classifier model was trained to distinguish between health-related and non-health-related web pages using the dataset created from the German health web. This model was tested for accuracy and precision on an 80/20 training/test split and against a crowd-validated dataset. For predicting cardiovascular diseases, the best-suited technique was the ‘Decision Tree’ compared with eight other techniques, i.e., Deep Learning, Nearest Neighbor (k-NN), Gradient Boosted Tree, Generalized Linear Model, Logistic Regression, Naïve Bayes, Random Forest, and Rule Induction [18]. Furthermore, some parameters were optimized using the optimized parameters operator to achieve better results when using the ‘Decision Tree’.

### 2.5. Data Model Validation and Testing

This step’s primary goal is to validate and test the selected model for the data in the model development process. The validation procedure is used to ensure that the developed model is accurate enough for the intended use [68]. The first half of this step, model validation, is important because the used/newly developed model cannot be relied on solely because it was designed to fit the training data and demonstrates that the training data fits the model well. To validate a model, output predictions are made in scenarios unrelated to the training set, and the same statistical measures of fit are computed. The second half of this step involves testing the model with test data and comparing its accuracy with the results of the validation step. Only when a model is compared to test data and statistical calculations show a satisfactory match is it considered “ready”. For the classification of tumor and non-tumor samples, Dong et al. [69] employed a training dataset (which consists of mass spectrometry (MS) raw data obtained from 194 paired tumor and non-tumor samples) to train different models and used a similar type of dataset (which consists of MS raw data obtained from 58 paired tumor and non-tumor samples) as a test dataset. The convolutional neural network (CNN), gradient boosting decision tree (GBDT), support-vector machine (SVM), principal component analysis (PCA) plus SVM, logistic regression (LR), and random forest (RF) were compared, and the CNN model showed the highest accuracy. Some of the ML model validation testing tools include Apache Spark, Excel, Hadoop, KNIME, Python, R, RapidMiner, SAS, SQL, and Tableau.

### 2.6. Evaluation

In the fifth phase (known as evaluation) of CRISP-DM, a more thorough evaluation and review of the model’s construction is conducted to ensure that the model properly achieves the business objectives. The steps in the evaluation phase are (1) the assessment of outcomes to assess how well the model achieves the project’s goals, discover additional constraints, information, or clues about future paths, and present the project’s final statement, (2) the review process to conduct a more in-depth review of the project and address quality assurance concerns, and (3) the decision for further steps to determine whether or not to proceed with the deployment or to make changes for the improvement [14].

After the analysis of text data, the next step is to visualize the data meaningfully for interpretation and communication purposes. Text visualization is primarily accomplished through the use of charts, graphs, maps, timelines, networks, word clouds, and so on. These visualized results allow humans to read the most important aspects of a large amount of information. There are several tools available to display the analyzed data. These tools make it easy to identify and discover patterns, outliers, trends, and insights in data straightforwardly and understandably. Effective data visualization has benefits and advantages such as easy understanding of the outcome, effortless and prompt decision-making, and a higher degree of engagement for a diverse audience over other communication methods (e.g., verbal communication). For successful data visualization, there are three main principles: (1) Depending on the purpose, select the appropriate visualization style, (2) the selected visualization style should be appropriate for the targeted audience, and (3) the chosen visualization style should be accompanied by an effective graphic design [70]. The most important aspects of selecting the appropriate visualization style are considering the selected data and the aim of the visualization. For example, line and bar charts are suitable for comparing data points across a dataset. Diverse visualization styles are available for creating attractive and effective visual information, i.e., typographic visualization (e.g., word cloud), graph visualization (e.g., tree), chart visualization (e.g., bar/line chat), 3D visualization, etc. Below, in Table 7, we provide a list of various visualization styles along with a few of the available tools in each category.

Besides these tools, there are software available with gigantic capabilities to visualize the data, such as, Microsoft Excel’s PivotTables, R, Tableau, Power-BI, datawrapper, and Google Charts. These tools are easy to use and very helpful in creating a clear and dynamic display of data because of their interactive graphical interface. Furthermore, different libraries written in different programming languages are also available for data visualization, which are easy to use for programmers, such as JavaScript libraries (e.g., D3.js, Chart.js, and Highcharts), python libraries (e.g., Matplotlib, Seaborn, and Plotly), and R libraries (e.g., ggplot2, Leaflet, and Esquisse). The major challenges of data visualization are the massive amount of data, the complexity of data, and missing/duplicate entries [124].

### 2.7. Deployment

In the deployment phase (sixth and final phase of CRISP-DM, Shearer [14]), the knowledge gained from the project is organized and presented (e.g., live demonstrations) in a way that is useful for the project, the company, and the customer. This phase’s complexity varies greatly. The steps in the deployment phase are as follows: (1) Create a deployment plan to formulate and note a deployment strategy for the model, (2) plan the monitoring and maintenance to create well-thought-out planning of maintenance and monitoring to shun problems during the operational phase of a model, (3) produce a final report to prepare and present a final report of the project in the form of a written document and verbal meeting, and (4) review the project to evaluate successes and failures, as well as potential areas for improvement in future projects.

## 3. Conclusions and Future Outlook

The amount of medical text data is rapidly increasing. From medical text data, data mining can be used to extract new and useful information or knowledge. The CRISP-DM system presented in this study focuses on each step of data mining while using medical examples to explain each step. The authors plan to develop an artificial intelligence-based web crawling system with 4D visualization of the data in a summarized and easy-to-understand manner and use these data as a source of information for researchers, as well as for the education of patients and medical staff in future work.

## Figures and Tables

**Table 1:** AI\ML products and research prototypes from some leading organizations in healthcare.

Products/Research Prototypes	Treatment/Field of Study	Company/Institution	Reference
MergePACS™	Clinical Radiology Imaging	IBM Watson	Merge PACS—Overview|IBM
BiometryAssist™	Diagnostic Ultrasound	Samsung Medison	https://www.intel.com/content/www/us/en/developer/tools/oneapi/application-catalog/full-catalog/diagnostic-ultrasound.html (accessed on 17 February 2022)
LaborAssist™	Diagnostic Ultrasound	Samsung Medison	
Breast Cancer Detection Solution	Ultrasound, mammography, MRI	Huiying’s solution	https://builders.intel.com/ai/solutionscatalog/breast-cancer-detection-solution-657 (accessed on 17 February 2022)
CT solution	Early detection of COVID-19	Huiying’s solution	https://builders.intel.com/ai/solutionscatalog/ct-solution-for-early-detection-of-covid-19-704 (accessed on 17 February 2022)
Dr. Pecker CT Pneumonia CAD System	Classification and quantification of COVID-19	Jianpei Technology	https://www.intel.com/content/www/us/en/developer/tools/oneapi/application-catalog/full-catalog/dr-pecker-ct-pneumonia-cad-system.html (accessed on 17 February 2022)

**Table 2:** Comparison between four text mining tools.

	Requests	Scrapy	Beautiful Soup	Selenium
What is it?	HTTP library for Python	Open-source web framework written in Python	python library	Open-source application framework tool and python library
Goal	Sending HTTP/1.1 requests using Python	Can crawl or scrape websites and extract the structured data and saves it Can also be used for a wide range of tasks, monitoring, and automated testing	Can parse the data and scrape the web pages Extract information from XML and HTML documents	Useful for web scraping websites that are JavaScript heavy
Ideal usage	Used for simple and low-level complex web scraping tasks	Framework used for complex web scraping or web crawling tasks. Used for large-scale projects	Used for smaller web scraping tasks Toolkit for searching through a document (XML or HTML) and extracting important information	Developed for web testing Used for test automation of web applications Scraping JavaScript-heavy websites Used for small-scale and low-level complex projects
Advantage	A simple way to retrieve data from URL Scraping data from web Allows to read, write, post, delete, and update the data for the given URL Extremely easy to deal with cookies and sessions	Portable library Runs on Linux, Windows, and Mac One of the faster scraping libraries Can extract websites much faster than other tools Consumes less memory and CPU usage Building a robust, and flexible application with different functions	Learning and mastering it is easy Community support is readily available to resolve issues.	Deals with the Core JavaScript-heavy website Can handle AJAX and PJAX requests
Selectors	None	JCSS and XPath	CSS	CSS and Xpath
Documentation	Detailed and simple to understand	Detailed and simple to understand	Detailed and simple to understand	Detailed and very complex
GitHub stars	46.8 k	42.7 k	-	22.7 k
Reference	Chandra and Varanasi [27]	Kouzis-Loukas [28]	Richardson [29]	Sharma [30]

**Table 3:** Databases and registries for clinical trials.

Databases/Registries	Trial Numbers	Provided by	Location	Founded Year	URL
ClinicalTrials.gov	405,612	U.S. National Library of Medicine	Bethesda, MD, USA	1997	https://clinicaltrials.gov/ (accessed on 11 April 2022)
Cochrane Central Register of Controlled Trials (CENTRAL)	1,854,672	a component of Cochrane Library	London, UK	1996	https://www.cochranelibrary.com/central (accessed on 11 April 2022)
WHO International Clinical Trials Registry Platform (ICTRP)	353,502	World Health Organization	Geneva, Switzerland	-	https://trialsearch.who.int/ (accessed on 11 April 2022)
The European Union Clinical Trials Database	60,321	European Medicines Agency	Amsterdam, The Netherlands	2004	https://www.clinicaltrialsregister.eu/ctr-search/search (accessed on 11 April 2022)
CenterWatch	50,112	-	Boston, MA, USA	1994	http://www.centerwatch.com/clinical-trials/listings/ (accessed on 11 April 2022)
German Clinical Trials Register (Deutsches Register Klinischer Studien—DRKS)	>13,000	Federal Institute for Drugs and Medical Devices	Cologne, Germany		https://www.bfarm.de/EN/BfArM/Tasks/German-Clinical-Trials-Register/_node.html (accessed on 11 April 2022)

**Table 4:** Research data in Medicine.

Databases	No. of Datasets	Owned by	Domains	Available Resources	URL	Ref
Biologic Specimen and Data Repository Information Coordinating Center (BioLINCC)	262	National Institute of Health, Calverton, MD, USA	Cardiovascular, pulmonary, and hematological	Specimens and Study Datasets	https://biolincc.nhlbi.nih.gov/studies/ (accessed on 4 April 2022)	[33]
Biomedical Translational Research Information System (BTRIS)	Five billion rows of data	Bethesda, MD, USA	Multiple subjects	Study Datasets	https://btris.nih.gov/ (accessed on 4 April 2022)	[34]
Clinical Data Study Request	3135	The consortium of clinical study Sponsors	Multiple subjects	Study Datasets	https://www.clinicalstudydatarequest.com/ (accessed on 4 April 2022)	[35]
Surveillance, Epidemiology, and End Results (SEER)	-	National Cancer Institute, Bethesda, MD, USA	Cancer (All types)—Stage and histological details	Study Datasets	https://seer.cancer.gov/ (accessed on 4 April 2022)	[36]
Medical Information Mart for Intensive Care (MIMIC) MIMIC-III	53,423 patients	MIT Laboratory for Computational Physiology, Cambridge, MA, USA	Intensive Care	Patient data (vital signs, medications, laboratory measurements, observations and notes charted by care providers, survival data, hospital length of stay, imaging reports, diagnostic codes, procedure codes, and fluid balance)	https://mimic.mit.edu/ (accessed on 4 April 2022)	[37,38]
MIMIC-CXR	65,379 patients (377,110 images of chest radiographs)					[39]
National Health and Nutrition Examination Survey (NHANES)	-	Centers for disease control and prevention, Hyattsville, MD, USA	Dietary assessment and other nutrition surveillance	data nutritional status, dietary intake, anthropometric measurements, laboratory tests, biospecimens, and clinical findings.	https://www.cdc.gov/nchs/nhanes/index.htm (accessed on 4 April 2022)	[40]
Global Burden of Disease (GBDx)	-	Institute for Health Metrics and Evaluation, Seattle, WA, USA	Epidemic patterns and disease burden	Surveys, censuses, vital statistics, and other health-related data	https://ghdx.healthdata.org/ (accessed on 4 April 2022)	[41]
UK Biobank (UKB)	0.5 million	Stockport, UK	In-depth genetic and health information	Genetic, biospecimens, and health data	https://www.ukbiobank.ac.uk/ (accessed on 4 April 2022)	[42]
The Cancer Genome Atlas (TCGA)	molecularly characterized over 20,000 cancer samples spanning 33 cancer types	National Cancer Institute, NIH, Bethesda, MD, USA	Cancer genomics	over 2.5 petabytes of epigenomic, proteomic, transcriptomic, and genomic data	https://www.cancer.gov/about-nci/organization/ccg/research/structural-genomics/tcga (accessed on 4 April 2022)	[43]
Gene Expression Omnibus (GEO)	4,981,280 samples	National Center for Bioinformatics (NCBI), NIH, Bethesda, MD, USA	Sequencing and gene expression	4348 datasets available	https://www.ncbi.nlm.nih.gov/geo/ (accessed on 4 April 2022)	[44]

**Table 5:** Biomedical literature sources.

Source	Articles (Million)	Launched by	Publication Type	Topic	Online	Link
PubMed	33	National Center for Biotechnology Information (NCBI)	Abstracts	Biomedical and life sciences	1996	https://www.ncbi.nlm.nih.gov/pubmed/ (accessed on 4 April 2022)
PubMed Central (PMC)	7.6	National Center for Biotechnology Information (NCBI)	Full text	Biomedical and life sciences	2000	https://www.ncbi.nlm.nih.gov/pmc/ (accessed on 4 April 2022)
Cochrane Library	-	Cochrane	Abstracts and full text	Healthcare	-	https://www.cochranelibrary.com/search (accessed on 4 April 2022)
bioRxiv	-	Cold Spring Harbor Laboratory (CSHL)	Unpublished preprints	Biological sciences	2013	https://www.biorxiv.org/ (accessed on 4 April 2022)
medRxiv	-	Cold Spring Harbor Laboratory (CSHL)	Unpublished manuscripts	Health sciences	2019	https://www.medrxiv.org/ (accessed on 4 April 2022)
arXiv	2.05	Cornell Tech	Non-peer-reviewed	Multidisciplinary	1991	https://arxiv.org/ (accessed on 4 April 2022)
Google Scholar	100 (in 2014)	Google	full text or metadata	Multidisciplinary	2004	https://scholar.google.com/ (accessed on 4 April 2022)
Semantic Scholar	205.25	Allen Institute for Artificial Intelligence	Abstracts and full text	Multidisciplinary	2015	https://www.semanticscholar.org/ (accessed on 4 April 2022)
Elsevier	17 (as of 2018)	Elsevier	Abstracts and full text	Multidisciplinary	1880	https://www.elsevier.com/ (accessed on 4 April 2022)
Springer Nature	-	Springer Nature Group	Abstracts and full text	Multidisciplinary	2015	https://www.springernature.com/ (accessed on 4 April 2022)
Springer	-	Springer Nature	Abstracts and full text	Multidisciplinary	1842	https://link.springer.com/ (accessed on 4 April 2022)

**Table 6:** Searching for relevant content.

	Natural Language Toolkit	SpaCy	Scikit-Learn NLP Toolkit	Gensim
What is it?	open-source python platform for handling human language data	open-source python library for advanced natural language processing	machine learning software library for the Python programming language	fastest python library for the training of vector embedding
Features			Based on NumPy, SciPy, and Matplotlib An easy and efficient way to analyze predictive data Easily accessible and reusable in different contexts	
Advantage	Most well-known and comprehensive NLP libraries with many extensions offers support in the largest number of languages	easy to use fully integrated with Python compatible with other deep learning frameworks many already trained statistical models available applicable to many different languages high speed and performance freely available able to process long texts platform-independent usable	simple and efficient tools for machine learning, data mining, and data analysis freely available for everyone applicable to different application areas, like natural language processing	Provides ready-to-use models and corpora Models pre-trained for specific areas such as health care Processes large amounts of data using streaming data
NLP Tasks	Classification Tokenization Stemming Tagging Parsing	Classification Tokenization Stemming Tagging Parsing Named Entity recognition Sentiment Analysis	Classification Topic Modeling Sentiment Analysis	Text similarity Text summarization Topic Modeling
GitHub stars	10.4 k	22.4 k	49 k	12.9 k
Website	nltk.org (accessed on 16 March 2022)	spacy.io (accessed on 16 March 2022)	scikit-learn.org (accessed on 16 March 2022)	radimrehurek.com/gensim/ (accessed on 16 March 2022)
Reference	Bird et al. [59]	Honnibal [60]	Pedregosa et al. [61], Pinto et al. [62]	Rehurek and Sojka [63]

**Table 7:** Data visualization style with exemplary tools.

Visualization Style	Tool [Reference]
Text marking/highlighting	cite2vec [71], TopicLens [72], SurVis [73], Poemage [74], Overview [75]
Tags or word cloud	SentenTree [76], InfoVis [77], VisOHC [78], IncreSTS [79], Word storms [80]
Bar charts	TextTile [81], SentiCompass [82], NewsViews [83], WeiboEvents [84], CatStream [85]
Scatterplot	PhenoLines [86], SocialBrands [87], TopicPanorama [88], #FluxFlow [89], PEARL [90]
Line chart	Vispubdata.org [91], GameFlow [92], MultiConVis [93], Contextifier [94], Google+Ripples [95]
Node-link	NEREx [96], iForum [97], NameClarifier [98], DIA2 [99], Information Cartography [100]
Tree	OpinionFlow [101], Rule-based Visual Mappings [102], HierarchicalTopics [103], Whisper [104], The World’s Languages Explorer [105]
Matrix	Interactive Ambiguity Resolution [106], Fingerprint Matrices [107], Conceptual recurrence plots [108], The Deshredder [109], Termite [110]
Stream graph timeline	VAiRoma [111], CiteRivers [112], ThemeDelta [113], EvoRiver [114], LeadLine [115]
Flow timeline	TimeLineCurator [116], Interactive visual profiling [117]
Radial visualization	ConToVi [118], ConVis [119]
3D visualization	Two-stage Framework [120]
Maps/Geo chart	Can Twitter save lives? [121], Visualizing Dynamic Data with Maps [122], Spatiotemporal Anomaly Detection [123]

## Data Availability

Not applicable.
